# Case Report: An unusual case of wide ileoileal intussusception associated with intestinal volvulus in a 8-months-old infant

**DOI:** 10.3389/fped.2024.1363731

**Published:** 2024-02-16

**Authors:** Giorgia Romano, Simone Frediani, Ivan Pietro Aloi, Arianna Bertocchini, Valerio Pardi, Antonella Accinni, Alessandro Inserra

**Affiliations:** Department of General Surgery, Bambino Gesù Children’s Hospital, IRCCS, Rome, Italy

**Keywords:** ileoileal intussusception, intestinal volvulus, children, abdominal pain, intussusception

## Abstract

**Introduction:**

Midgut volvulus and intussusception are prevalent paediatric abdominal emergencies. To the best of our knowledge, this is the first reported case of a connection between intestinal volvulus and a massive intussusception.

**Case report:**

An 8-month-old male infant was brought to the emergency room with a history of abdominal pain and vomiting for <24 h. On physical examination, the child appeared restless and was found to have a circumferential hard mass of approximately 4 cm in diameter in the epigastric region. Upon admission, laboratory results showed a C-reactive protein level of 0.4 mg/dl, LDH level of 351 U/L, mild leukocytosis with a white blood cell count of 12 × 103 /µl, and 67% neutrophils. A physical exam was significant for abdominal distention, hyperresonance in percussion, and a palpable, painful epigastric mass. The findings of the operation included a dilated and ischemic intestinal loop, approximately 25 cm from the ileocecal valve, twisted upon itself for three turns. After de-rotation, an extensive occluding ileo-ileal invagination with an ischemic intestinal loop was identified, and a length of approximately 55–60 cm of the distal ileum, including the ischemic segment, was resected.

**Discussion:**

This is the first reported case of a connection between intestinal volvulus and a massive intussusception. Currently, only two reported cases describe the connection between volvulus and intussusception, which are insufficient to establish a direct link between the two clinical conditions.

## Introduction

Midgut volvulus and intussusception are two common abdominal surgical emergencies in children under the age of 3 years. Intussusception occurs when a proximal segment of the intestine, or intussusceptum, invaginates into a distal segment of the intestine, or intussuscipiens. The associated mesentery is dragged within the invaginated segment, leading to venous congestion and oedema. This may result in ischemia and eventually bowel necrosis, perforation, and peritonitis, if left untreated (1). Air enema reduction techniques monitored by ultrasonography are the preferred first-line treatment, with good results and few complications. When bowel necrosis or perforation is suspected or non-operative treatment with enema reduction fails, surgical reduction is indicated as an open or laparoscopic procedure (2, 3). In hemodynamically stable children with intussusception, without critical illness, non-operative outpatient management should be maximized and minimally invasive techniques are to be preferred to avoid laparotomy (4, 5). Intestinal malrotation is a congenital anomaly that, when associated with midgut volvulus, requires an emergent operation to prevent or limit the degree of intestinal ischemia and the need for small bowel resection. Intestinal rotation abnormalities constitute a spectrum of conditions that arise from perturbations in the normal embryological process of herniation, rotation, and fixation of the midgut. The failure of this process results in midgut malrotation; the exact cause is not yet established. However, there is often a fibrous band, the Ladd's band, that tethers the right colon, and prevents it from rotating. Midgut malrotation may be asymptomatic, but can also lead to the most serious form of obstruction: the midgut volvulus (6). The bowel loops twist around the mesenteric artery or its branches, resulting in variable degrees of ischemic necrosis (7). The curative surgical treatment is the Ladd procedure, which involves untwisting the intestine, surgical division of any Ladd's bands between the caecum and the duodenum, widening of the mesenteric root to prevent further volvulus, positioning of the intestines into the non-rotation configuration and often appendectomy to prevent future diagnostic confusion (8). Surgeons can perform the procedure through a midline incision or laparoscopy. Both intestinal volvulus and intussusception can escalate and lead to life-threatening complications; therefore, a high index of suspicion and thorough knowledge of these conditions are of major importance for all surgeons. To the best of our knowledge, this is the first case of association between intestinal volvulus and such extensive intussusception.

## Case report

**Figure 1:**
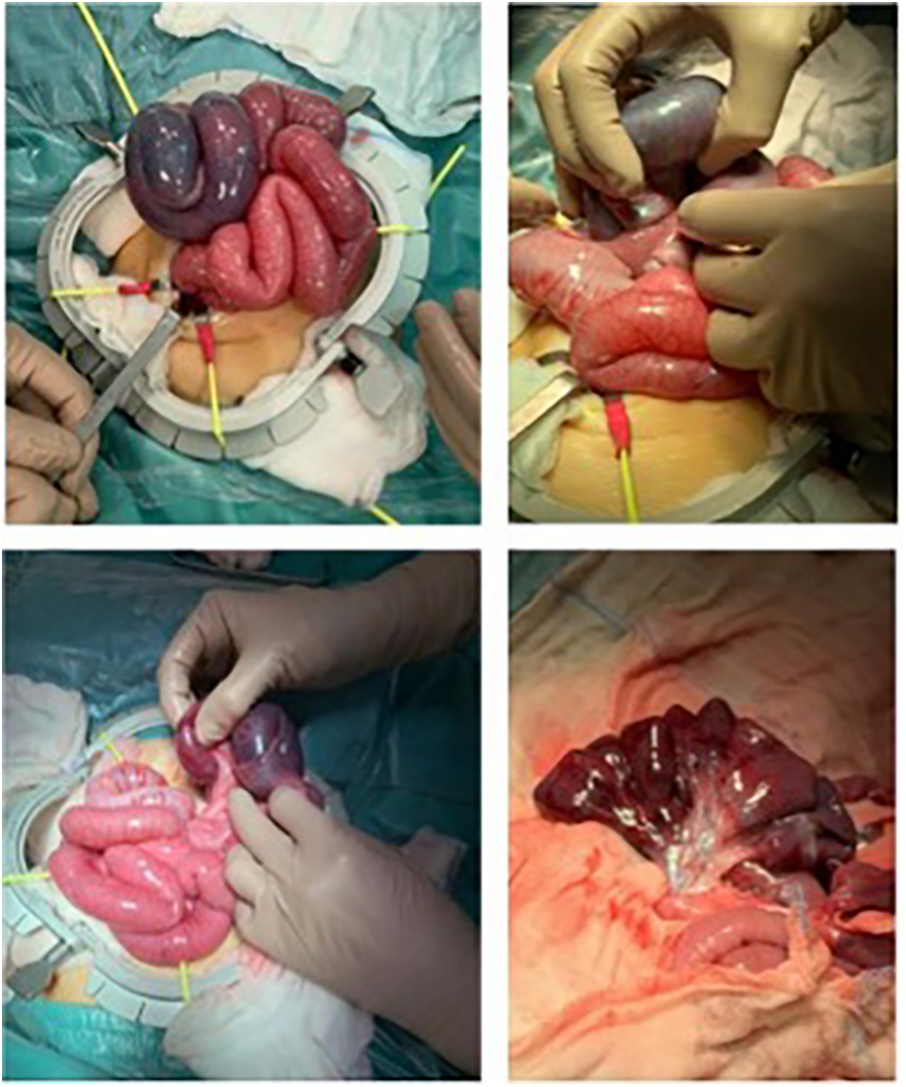
The findings of the operation included a dilated and threatened intestinal loop, about 25 cm from the ileocecal valve, twisted upon itself for 3 turns. After de-rotation, an extensive occluding ileo-ileal invagination with an ischemic intestinal loop was identified, and approximately 55–60 cm of the distal ileum, including the ischemic segment, were resected.

An 8-month-old male infant was brought to the emergency room with a history of abdominal pain and vomiting for <24 h. The last evacuation of normally formed greenish stools was 24 h prior to the patient's presentation. The patient was born full-term (40 + 2 weeks gestational age) and healthy, via spontaneous vaginal delivery and the medical history was unremarkable with no previous surgeries or hospitalization. On physical examination, the patient appeared restless and was observed to have a circumferential mass of hard consistency, of approximately 4 cm in diameter, in the epigastric region. Upon admission, laboratory results showed a C-reactive protein level of 0.4 mg/dl, LDH level of 351 U/L, mild leukocytosis with a white blood cell count of 12 × 103 /µl, and 67% neutrophils. The remaining lab values showed no abnormalities, with electrolyte levels within normal range. A physical exam was significant for abdominal distention, hyperresonance in percussion, and a palpable painful epigastric mass, which is an infrequent finding, being encountered in 7.5%–40% of children with intussusception (9). An abdominal ultrasound revealed the presence of a long, presumably ileal loop convoluted upon itself for several turns in the central abdominal quadrants. The loop had thickened and had hypoperistaltic walls, with no vascularity detected on colour Doppler. It also showed evident free intra-abdominal fluid. Plain abdominal radiography confirmed the absence of intestinal meteorism, likely due to the ileal tangle. There was no air in the rectal ampulla and no definitive signs of free air. In consideration of the patient's clinical condition and the radiological suspicion of an acute abdomen due to a probable volvulus in the setting of possible ileoileal intussusception, urgent surgical exploration was recommended. An exploratory laparotomy was carried out, with a medial hyper infraumbilical incision and access to the abdominal cavity. The findings of the operation included a dilated and threatened intestinal loop, approximately 25 cm from the ileocecal valve, twisted upon itself for three turns. After de-rotation, an extensive occluding ileo-ileal invagination with an ischemic intestinal loop was identified, and approximately 55–60 cm of the distal ileum, including the ischemic segment, were resected (Figure 1). An intramural 8 mm lymph node was also found in close proximity to the invaginated loop and was excised. The rest of the bowel up until the ligament of Treitz showed no malrotation nor sign of hypoperfusion. An end-to-end ileo-ileal anastomosis was performed hand-sewn and an abdominal drainage was placed in the right iliac fossa. Postoperative recovery of the patient was uneventful and the patient showed progressive recovery of physiological functions with the return of regular bowel function on the third postoperative day. The resumption of normal oral feeding took place on the fifth postoperative day and was well tolerated. The medical team discharged the patient on the seventh postoperative day, subject to the removal of the abdominal drain. Thus far, the patient has had no relapses and no symptoms related to intestinal occlusion.

## Discussion

The pathophysiology of the majority of paediatric intussusception cases is thought to be secondary to a transient viral illness leading to temporary lymphatic engorgement, creating a lead point and resultant intussusception (10). In our patient, intestinal intussusception may have been consequent to the volvulus. A congenital anomaly in embryonic development may have caused intestinal malrotation with the volvulus, leading to lymphatic stasis, inflammation of loops, and consequently creating conditions for the development of intussusception. Bowel obstruction caused by the volvulus also results in distress to the intestinal walls, release of inflammatory mediators, and increased oxidative stress. All these factors contribute to an alteration in the permeability of the intestinal barrier, promoting the pathological migration of infectious agents, and eventually potential infections associated with lymphoid hyperplasia—an additional hypothetical cause of intussusception. It is equally plausible that the two pathological conditions are not causally linked. Both processes, favour and potentially cause intestinal occlusion with ischemic distress, lymphatic and blood stasis, and loop distension. A vicious cycle may have formed, causing the development of the rare association observed in this clinical case. To our knowledge, this is the first documented case of association between intestinal volvulus and extensive intussusception. Currently, given the paucity of reported cases, there are no scientific data to support the direct correlation between the two pathological entities. There are only two other reported cases describing an association between volvulus and intussusception. A previous study recounts a case of foetal intestinal volvulus associated with type 3A jejunal atresia and on the distal end of the atresia, a 57 cm segment of small bowel-to-small bowel intussusception; intestinal pathology was noted on prenatal ultrasound (11). Another case represents an unusual complication of Roux-en-y gastric bypass surgery in a 22-year-old woman involving intussusception, internal hernia, and volvulus that was successfully managed without the need for bowel resection due to early identification and surgical intervention (12). Rapid surgical treatment is key to preventing bowel infarctions necessitating resection, which can lead to short gut syndrome, adhesions, and other complications (13). A laparoscopic method, which is associated with lesser post-operative pain, a quicker time to reach full feed, lesser scarring, and fewer post-operative problems than that in a laparotomy, can be used to treat intestinal malrotation, if carried out by a trained surgical team. The major concern with the laparoscopic treatment of malrotation is the risk of recurrent midgut volvulus and the age for the limited working space and vision. As laparoscopy creates fewer adhesions than that in a laparotomy, it is believed to be associated with a higher risk of recurrent midgut volvulus. Furthermore, if midgut volvulus has caused intestinal necrosis, conversion to laparotomy and bowel resection are still required (14, 15).

## Data Availability

The original contributions presented in the study are included in the article/Supplementary Material, further inquiries can be directed to the corresponding author.

## References

[1] Charles T Penninga L Reurings JC Berry MC. Intussusception in children: a clinical review. Acta Chir Belg. (2015) 115(5):327–33. 10.1080/00015458.2015.11681124 26559998

[2] Edwards EA Pigg N Courtier J Zapala MA MacKenzie JD Phelps AS. Intussusception: past, present and future. Pediatr Radiol. (2017) 47(9):1101–8. 10.1007/s00247-017-3878-x 28779197

[3] Hill SJ Koontz CS Langness SM Wulkan ML. Laparoscopic versus open reduction of intussusception in children: experience over a decade. J Laparoendosc Adv Surg Tech A. (2013) 23(2):166–9. 10.1089/lap.2012.0174 23327343

[4] Benedict LA Ha D Sujka J Sobrino JA Oyetunji TA St Peter SD The laparoscopic versus open approach for reduction of intussusception in infants and children: an updated institutional experience. J Laparoendosc Adv Surg Tech A. (2018) 28(11):1412–5. 10.1089/lap.2018.0268 30036131

[5] Kia KF Mony VK Drongowski RA Golladay ES Geiger JD Hirschl RB Laparoscopic vs open surgical approach for intussusception requiring operative intervention. J Pediatr Surg. (2005) 40(1):281–4. 10.1016/j.jpedsurg.2004.09.026 15868598

[6] Langer JC. Intestinal rotation abnormalities and midgut volvulus. Surg Clin North Am. (2017) 97(1):147–59. 10.1016/j.suc.2016.08.011 27894424

[7] Eltayb AB Hegazi A Elhag O Abdelgadir A. Midgut volvulus due to congenital malrotation in an adult: a case report. J Med Case Rep. (2023) 17(1):378. 10.1186/s13256-023-04096-5 37620962 PMC10463942

[8] Shalaby MS Kuti K Walker G. Intestinal malrotation and volvulus in infants and children. Br Med J. (2013) 347:f6949. 10.1136/bmj.f6949 24285798

[9] Waseem M Rosenberg HK. Intussusception. Pediatr Emerg Care. (2008) 24(11):793–800. 10.1097/PEC.0b013e31818c2a3e 19018227

[10] Kelley-Quon LI Arthur LG Williams RF Goldin AB St Peter SD Beres AL Management of intussusception in children: a systematic review. J Pediatr Surg. (2021) 56(3):587–96. 10.1016/j.jpedsurg.2020.09.055 33158508 PMC7920908

[11] Ohuoba E Fruhman G Olutoye O Zacharias N. Perinatal survival of a fetus with intestinal volvulus and intussusception: a case report and review of the literature. AJP Rep. (2013) 3(2):107–12. 10.1055/s-0033-1349367 24147247 PMC3799706

[12] Elkbuli A Santarone K Kinslow K McKenney M Boneva D. A rare case of internal hernia, intussusception and volvulus following gastric bypass: a case report and literature review. Int J Surg Case Rep. (2020) 67:178–82. 10.1016/j.ijscr.2020.01.060 32062127 PMC7021521

[13] Ayane GN Kadimo K. Diagnosis and surgical management of congenital intestinal malrotation presenting with midgut volvulus in an adult: high index of suspicion (case report). Pan Afr Med J. (2018) 29:154. 10.11604/pamj.2018.29.154.13910 30050618 PMC6057601

[14] Svetanoff WJ Srivatsa S Diefenbach K Nwomeh BC. Diagnosis and management of intestinal rotational abnormalities with or without volvulus in the pediatric population. Semin Pediatr Surg. (2022) 31(1):151141. 10.1016/j.sempedsurg.2022.151141 35305800

[15] Scalabre A Duquesne I Deheppe J Rossignol G Irtan S Arnaud A Outcomes of laparoscopic and open surgical treatment of intestinal malrotation in children. J Pediatr Surg. (2020) 55(12):2777–82. 10.1016/j.jpedsurg.2020.08.014 32972740

